# Motor tract lesion mapping from the brain to the lower spinal cord in people with relapsing–remitting multiple sclerosis: exploring the association between lesion severity and functional consequences by limb

**DOI:** 10.1093/braincomms/fcag140

**Published:** 2026-04-17

**Authors:** Malo Gaubert, Alice Dufey, Elise Bannier, Benoît Combès, Audrey Rico, Jean-Christophe Ferré, Raphaël Chouteau, Paul Sauleau, Guillaume Hamon, Mathilde Liffran, Laure Michel, Emmanuelle Le Page, Virginie Callot, Bertrand Audoin, Sarah Demortière, Anne Kerbrat

**Affiliations:** Service de Radiologie et Imagerie Médicale, Univ Rennes, CHU Rennes, Rennes F-35000, France; Empenn Research Unit U1228, Univ Rennes, Inria, CNRS, Inserm, IRISA UMR 6074, Rennes F-35000, France; Service de Neurologie, Univ Rennes, CHU Rennes, Rennes F-35000, France; Service de Radiologie et Imagerie Médicale, Univ Rennes, CHU Rennes, Rennes F-35000, France; Empenn Research Unit U1228, Univ Rennes, Inria, CNRS, Inserm, IRISA UMR 6074, Rennes F-35000, France; Empenn Research Unit U1228, Univ Rennes, Inria, CNRS, Inserm, IRISA UMR 6074, Rennes F-35000, France; Assistance Publique-Hopitaux de Marseille (AP-HM), Hôpital Universitaire Timone, CEMEREM, F-13385 Marseille, France; CRMBM, Aix-Marseille University, CNRS,F-13385 Marseille, France; Department of Neurology, Assistance Publique-Hopitaux de Marseille (AP-HM), Hôpital Universitaire Timone, F-13385 Marseille, France; Service de Radiologie et Imagerie Médicale, Univ Rennes, CHU Rennes, Rennes F-35000, France; Empenn Research Unit U1228, Univ Rennes, Inria, CNRS, Inserm, IRISA UMR 6074, Rennes F-35000, France; Service de Neurologie, Univ Rennes, CHU Rennes, Rennes F-35000, France; Service des Explorations Fonctionnelles, Univ Rennes, CHU Rennes, Rennes F-35000, France; Service de Radiologie et Imagerie Médicale, Univ Rennes, CHU Rennes, Rennes F-35000, France; Empenn Research Unit U1228, Univ Rennes, Inria, CNRS, Inserm, IRISA UMR 6074, Rennes F-35000, France; Service de Neurologie, Univ Rennes, CHU Rennes, Rennes F-35000, France; Service de Neurologie, Univ Rennes, CHU Rennes, Rennes F-35000, France; Assistance Publique-Hopitaux de Marseille (AP-HM), Hôpital Universitaire Timone, CEMEREM, F-13385 Marseille, France; CRMBM, Aix-Marseille University, CNRS,F-13385 Marseille, France; Assistance Publique-Hopitaux de Marseille (AP-HM), Hôpital Universitaire Timone, CEMEREM, F-13385 Marseille, France; CRMBM, Aix-Marseille University, CNRS,F-13385 Marseille, France; Department of Neurology, Assistance Publique-Hopitaux de Marseille (AP-HM), Hôpital Universitaire Timone, F-13385 Marseille, France; Assistance Publique-Hopitaux de Marseille (AP-HM), Hôpital Universitaire Timone, CEMEREM, F-13385 Marseille, France; CRMBM, Aix-Marseille University, CNRS,F-13385 Marseille, France; Department of Neurology, Assistance Publique-Hopitaux de Marseille (AP-HM), Hôpital Universitaire Timone, F-13385 Marseille, France; Empenn Research Unit U1228, Univ Rennes, Inria, CNRS, Inserm, IRISA UMR 6074, Rennes F-35000, France; Service de Neurologie, Univ Rennes, CHU Rennes, Rennes F-35000, France

**Keywords:** quantitative MRI, spinal cord, multiple sclerosis, motor evoked potentials, motor deficit

## Abstract

Motor deficits in people with multiple sclerosis (pwMS) are often asymmetrical, suggesting an important role of focal lesions affecting the corresponding motor pathways. However, only a modest relationship has been established between lesion load and physical disability. One hypothesis could be that only heavily demyelinated lesions along the corticospinal tract (CST) would be associated with functional consequences. To test this hypothesis, we reconstructed the whole CST from the cortex to the bottom of the spinal cord and linked its structural damage with its functional consequences as measured clinically [with the American Spinal Injury Association motor (mASIA) score] and electrophysiologically [with the central motor conduction time (CMCT)] by limb. We prospectively included 60 relapsing–remitting pwMS and 33 healthy controls. The CSTs were reconstructed using probabilistic atlases. Lesion volume fraction and myelin content [approximated through the magnetization transfer ratio (MTR) and quantitative T1 (qT1)] were calculated by side on the different portions of the CST. Voxelwise MTR z-score maps were also produced to detect lesions severely demyelinated (z-score < −1.96 SD) along the whole CST. Forty-six pwMS and 28 healthy controls were included in the analyses. In the upper limb, CMCT was associated with both cervical lesion load (P < 0.001) and cervical MTR (P = 0.02) in the CST. In the lower limb, CMCT was associated with cervical lesion load and brain and thoracic MTR (all P < 0.001) in the CST. The Expanded Disability Status Scale score was associated with brainstem lesion load and thoracic MTR (all P < 0.01) in the CST. No association was found with the mASIA score per limb. Twenty-six per cent of the lesions were classified as severe, mainly in the cervical CST. In patients with at least one severe lesion, extra-lesional MTR along the CST was lower than in those without severe lesions (P < 0.001). The presence of a severe lesion in the spinal cord CST was the only explanatory variable associated with an increased risk of having an abnormal lower limb CMCT (odds ratio [95% confidence interval]: 1.74 [1.36–2.24], P < 0.001). The main results were replicated with qT1. In this study, we describe varying levels of microstructural damage in lesions along the CST, with associations observed between lesion severity and both extra-lesional damage and CMCT. These findings highlight the critical role of severe lesions along the CST—particularly at the cervical level—beyond overall lesion load, and underscore the need to develop therapies that promote remyelination and to evaluate their effects at the cervical spinal cord level.

## Teaser

Using quantitative MRI, Gaubert *et al*. describe varying levels of demyelination in lesions along the brain and spinal cord motor tracts in people with relapsing–remitting multiple sclerosis. They observed associations between lesion severity and both extra-lesional damage and motor conduction deficits in the upper and lower limbs.

## Graphical Abstract

**Graphical Abstract:**
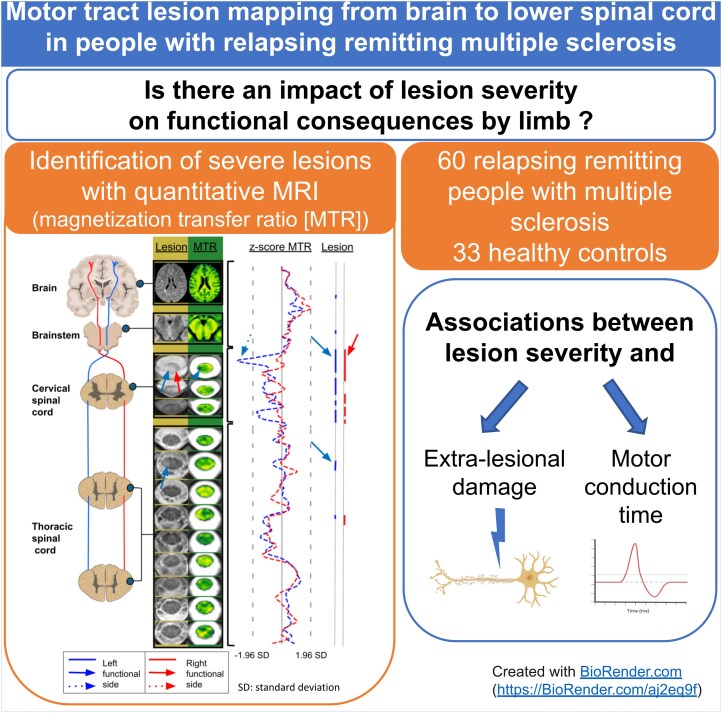

## Introduction

From a clinical perspective, motor deficits are common in people with multiple sclerosis (pwMS) and are a source of significant functional disability. These deficits can occur either following a relapse or independently of relapses.^1^ In both cases, the motor deficit is often asymmetrical or even exclusively affects one limb or one hemicorpus, suggesting an important role of focal lesions affecting the corresponding motor pathways.^2-4^

Several imaging studies of pwMS have focused on corticospinal tracts (CSTs), the main motor pathways. Brain and cervical spinal cord (SC) lesion load in the CST has been associated with motor disability, with a predominant role of infra-tentorial and SC lesions.^5-7^ Cortical lesions in the primary sensory–motor area of the hand have been linked to contralateral hand loss of sensory–motor function assessed by clinical measures and electrophysiology.^8^ However, the relationship between motor pathway lesion load and physical disability of pwMS remains uncertain.

Previous investigations have been restricted to the motor pathways of the brain,^5,8-12^ the cervical SC^13-15^ or the combination of brain and cervical SC^6,7^ and have rarely included the thoracic SC portion.^16^ Motor deficits, however, are generally predominant in the lower limbs. Moreover, multiple sclerosis lesions are heterogeneous, with different degrees of demyelination.^17^ One hypothesis is that particularly severe lesions (i.e. heavily demyelinated along the motor pathways) have functional consequences, while less severe or remyelinated lesions have no functional consequences. Testing this hypothesis requires the ability to quantify the severity of multiple sclerosis lesions in the motor tract *in vivo*. The severity of demyelination in multiple sclerosis lesions could be quantified in the brain using PET imaging^18^ or approximated using different quantitative MRI techniques in both the brain and the SC. The magnetization transfer ratio (MTR) can be used to approximate myelin content in the central nervous system.^19^ Similarly, quantitative T1 (qT1) mapping with the magnetization-prepared 2 rapid gradient echo (MP2RAGE) sequence allows demyelinated and remyelinated lesions to be differentiated.^20^ The MP2RAGE sequence, the acquisition of which is currently limited to the brain and cervical SC, has the advantage of being relatively fast and compatible with clinical routine acquisition.^21^ Finally, electrophysiology can also be used to approximate myelin content. The prolongation of central motor conduction time (CMCT) has been associated with demyelination^22^ and is sensitive to subclinical motor impairment.^23^

Taking into account these different observations, we propose to (i) describe the structural damage (lesion load and myelin content approximated using MTR) in the different portions of the CST from the top of the brain to the bottom of the thoracic SC in a cohort of people with relapsing–remitting multiple sclerosis (RRMS); (ii) study the relationship between the structural damage of the CST by side and its function as measured clinically and electrophysiologically by limb; (iii) describe the individual profiles of demyelination along the CSTs at the voxel level to identify highly demyelinated lesions and test their association with motor function; and (iv) reproduce the results obtained using MTR with the faster qT1 mapping for the brain and cervical portion of the CST.

## Materials and methods

### Study design

We carried out a prospective, cross-sectional, bicentric study.

### Participants

The MS-TRACTS study included 60 people with relapsing–remitting multiple sclerosis from Rennes and Marseille University hospitals between February 2021 and May 2023. Participants underwent MRI, electrophysiological assessments and clinical evaluations on the same day. The study was approved by the Institutional Review Board and registered on Clinical Trials (NCT04220814). The main inclusion criteria were (i) age over 18 years; (ii) relapsing–remitting multiple sclerosis diagnosis according to the 2017 revised criteria^24^; and (iii) having at least one clinical sign of central motor tract damage defined as motor deficit and/or hyperreflexia and/or Babinski sign at clinical examination. The main exclusion criteria were (i) relapse or corticosteroids in the month before inclusion; (ii) history of other neurological or systemic disease; and (iii) contraindications to MRI or motor evoked potentials (MEPs). Thirty-three age- and sex-matched healthy controls (HCs) with no history of neurological disorder were included for MRI assessment. Nine of them also had MEP evaluation. All participants gave written informed consent. Of the 60 pwMS and 33 HCs initially included in the study, 46 pwMS (*n* = 35/11, Rennes/Marseille) and 28 HCs (*n* = 21/7) were included in the analyses. Reasons for exclusion are summarized in the study flow-chart (Supplementary Fig. S1).

### Clinical assessment

The clinical assessment was performed by a senior neurologist in both centres. It included Expanded Disability Status Scale (EDSS), the pyramidal functional system score (FSS),^25^ the timed 25-foot walk (T25-FW),^26^ the 12-item Multiple Sclerosis Walking Scale (MSWS-12),^27^ the nine-hole peg test (9HPT),^28^ the French-language version of the urinary disorder-specific quality of life questionnaire^29^ and the American Spinal Injury Association motor (mASIA) score for each limb.^30^ The mASIA score is based on a manual strength test according to the MRC (Medical Research Council) scale for five muscles in the upper limb and five muscles in the lower limb. It is rated out of 25 for each limb, and scores <25 are defined as deficient. Lower scores reflect higher motor deficiency of the limb.

### Electrophysiological assessment

MEPs were acquired on the abductor digiti minimi muscle for the upper limbs and on the tibialis anterior muscle for the lower limbs. CMCT was calculated with the motor root stimulation technique. Neurophysiological recordings and measurements are detailed in Supplementary Text S1.

### MRI acquisition

Both recruiting centres were equipped with a 3 T MRI scanner (Magnetom Prisma in Rennes, VE11C; Magnetom Vida in Marseille, XA20) and a 64-channel head/neck coil from the same manufacturer (Siemens Healthineers, Erlangen, Germany). The same MR sequences and acquisition parameters were applied in both centres. Details of the acquisition parameters are given in Supplementary Table S1. In summary, brain sequences included a 3D fluid-attenuated inversion recovery (FLAIR), a 3D T1 magnetization-prepared rapid gradient echo (MPRAGE) and 3D gradient echo sequences with (MT1) and without (MT0) prepulse. SC sequences included two axial T2*w multigradient echo (MGE) sequences covering the cervical levels from C1 to C3 and from C4 to C7, two axial T2w turbo spin echo (TSE) sequences covering both thoracic and lumbar levels from C7 to T8 and from T8 to L1, a sagittal T2w TSE sequence covering the whole spine (split into two successive acquisitions), a sagittal T2 short TI inversion recovery (STIR) and 3D gradient echo sequences with (MT1) and without (MT0) prepulse divided into three successive acquisitions covering the whole SC. To minimize B1+ bias on MTR measures as previously reported,^31^ standardized positions of the slab centres of MT acquisitions were used (C5, T5 and T9–T10 vertebral levels). Finally, a 3D MP2RAGE sequence and 2D turbo FLASH B1+ map both covering the brain and the cervical SC from C1 to C7 were acquired.^32^ A composite volume, namely ‘uniform’ T_1_-weighted image (MP2RAGE_UNI_), used to derive a qT1 map, was automatically generated on-line by the scanner based on the 3D gradient recalled echo volumes acquired with the two inversion times.^33^ The B1+ map (flip angle map), used subsequently to correct the T1 map from B1+ inhomogeneities, was also automatically generated by the scanner.

### MRI processing

**Figure 1:**
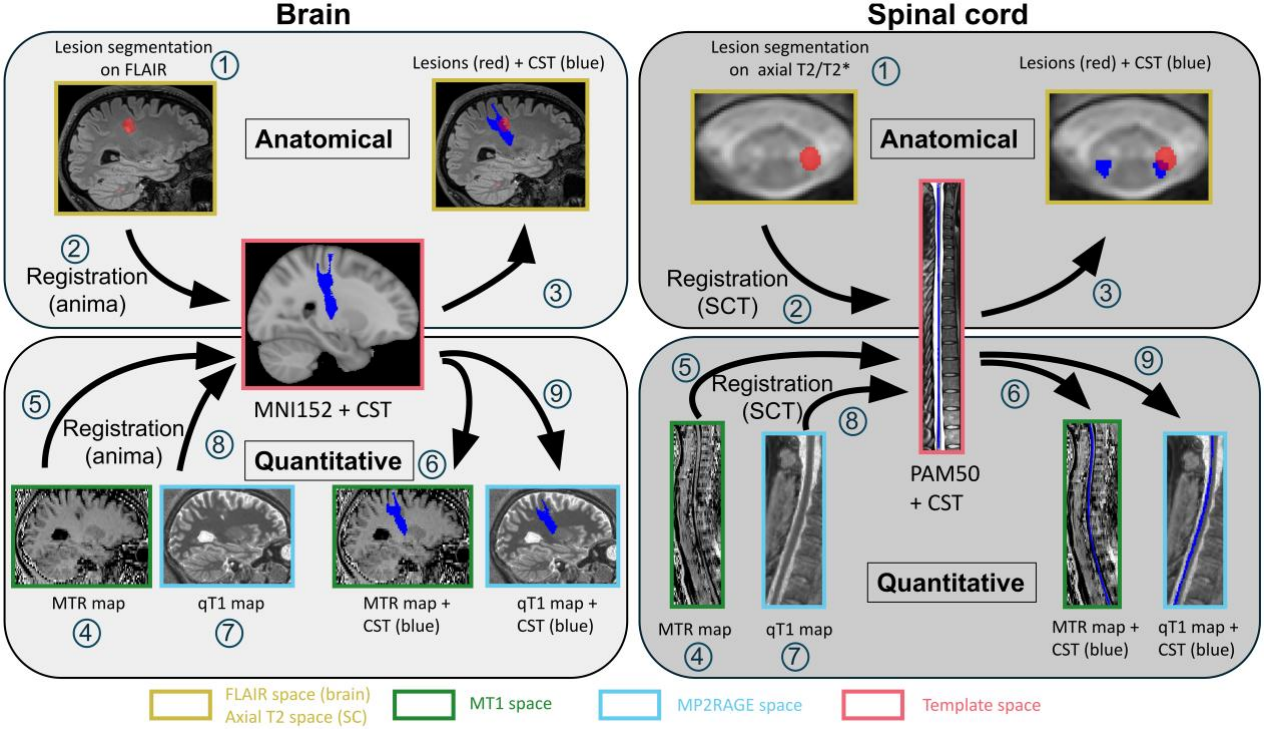
**Schematic view of brain (left) and SC (right) processing steps.**  Brain: lesion delineation on FLAIR image (1); computation of registrations from FLAIR (2), MT1 (5) and MP2RAGE (8) to MNI152 space (through T1w, except for MP2RAGE); linear registration of MT0 image to MT1 space and MTR map computation (4); qT1 map computation (7); application of inverse transformations (computed in 2, 5, 8) to register corticospinal tracts from MNI152 space to individual space (3, 6, 9). SC: lesion delineation on axial T2*w images (1); computation of registrations from axial T2*w (2), MT1 (5) and MP2RAGE (8) to PAM50 space through sagittal T2; linear registration of MT0 images to MT1 space and MTR maps computation (4); qT1 map computation (7; same map as for the brain); application of inverse transformations (computed in 2, 5, 8) to register corticospinal tracts from PAM50 space to individual space (3, 6, 9). On both brain and SC acquisitions, red colour shows a lesion, while blue colour shows the corticospinal tract. CST = corticospinal tract; FLAIR = 3D fluid-attenuated inversion recovery; MNI = Montreal Neurological Institute; MP2RAGE = magnetization-prepared 2 rapid acquisition gradient echoes; MTR = magnetization transfer ratio; PAM = Polytechnique, Aix-Marseille University and Montreal Neurological Institute; SC = spinal cord; qT1 = MP2RAGE quantitative T1. Alt text: figures depicted the image inputs and ouputs and arrows with number the processing step of anatomical and quantitative processing of brain and spinal cord images.

The main processing was performed using the Spinal Cord Toolbox (SCT) v6.1 for the SC images^34^ and the FMRIB Software Library (FSL) v6.0.5^35^ and Anima v4.1 (https://anima.irisa.fr, RRID:SCR_017072) for the brain and further processing. Visualization and lesion segmentation were performed using ITK-SNAP v3.8.0^36^ and FSLeyes (part of FSL). A thorough description of the processing steps is given in Fig. 1 and in Supplementary Text S2 for a description of the computation of brain volumes and SC cross-sectional areas.

#### Lesion delineation

Brain lesions were delineated on 3D FLAIR images using an in-house automatic lesion-detection tool, LongiSeg4ms (https://gitlab.inria.fr/amasson/longiseg4ms). Visual assessment was performed by two neurologists, and manual corrections were performed using ITK-SNAP when needed. SC lesions were manually delineated on the axial T2*w images for the cervical SC and axial T2w images for the thoracic SC by a neurologist using ITK-SNAP. MP2RAGE and STIR sequences for the upper part and sagittal T2w sequences for the whole SC were used to help identify the lesion.^37^ Of note, neither MTR maps nor MT images were used for lesion detection.

#### Magnetization transfer ratio computation

After a non-linear registration of the MT0 map to MT1 space (*animaPyramidalBMRegistration* for the brain, *sct_register_multimodal* for the SC), MTR maps were computed for the brain and the three SC levels using the following formula (*sct_compute_mtr)*:


$$MTR\;=\frac{MT0\;-\;MT1}{MT0}\times 100\;$$

#### Quantitative T1 computation

To reduce the B1+ bias that may exist through the image volume in the MP2RAGE_UNI_, which would lead to inaccurate T1 estimation if not considered, qT1 maps were generated by integrating the Bloch equations and a look-up-table approach accounting for B1+ variations.^38^

### Data analyses

#### Registration to template

The parameters of the transformation between native images and templates were computed in the brain and the SC for each modality (axial T2/T2*, MT, MP2RAGE). They were used either to warp back CSTs from atlases to individual space for region of interest (ROI)-based analyses or to register individual lesion masks and quantitative maps (MTR, qT1) to a common template for voxelwise *z*-score analyses.

##### Brain registration

T1w images were registered to MNI152 space using affine (*animaPyramidalBMRegistration*) and non-linear (*animaDenseSVFBMRegistration*) registrations. FLAIR images were rigidly registered to T1w space. Based on the merging of these two registrations, the transformations between FLAIR space (lesion) and MNI152 space were computed. Transformations from MT1 space to MNI152 space through T1w space were computed the same way as for FLAIR. An affine registration followed by a non-linear registration was also computed from MP2RAGE_UNI_ to MNI152 space.

##### Spinal cord registration

Following the SCT guideline, two-step models were used to register all modalities of interest to PAM50 space. First, the SCs were automatically segmented on sagittal T2 images (*sct_deepseg_sc*) and all vertebrae automatically (or manually when needed) labelled (*sct_label_vertebrae*). Then, the registration transformations between individual space and PAM50 were computed (*sct_register_to_template*). Second, using these parameters as initialization, registrations between each modality (axial T2/T2*, MT, MP2RAGE) and PAM50 space were calculated. Before these registrations, the SCs were segmented in all four axial T2/T2*, all three MT1 levels and MP2RAGE qT1 (*sct_deepseg*). All T2/T2* images (and associated lesion and SC masks) were gathered in one image (*animaImageMosaicing*). The same stitching was applied for the three MT1 levels (and associated SC and MTR maps). Finally, transformations from each modality (axial T2/T2*, MT1 and MP2RAGE qT1_I_) to PAM50 space were calculated (*sct_register_multimodal*).

#### ROI-based analyses

For these analyses, left and right CSTs were registered back from MNI152 or PAM50 space to each modality space (FLAIR, axial T2/T2*, brain/SC MT and brain/SC MP2RAGE qT1) for all HCs and pwMS from Rennes and Marseille to perform extraction in the native space. The CST ROIs were defined in the brain based on the left and right M1 cortex in the MNI152 template space, including both brain^39^ and brainstem parts,^40^ as previously described,^6^ and in the SC based on the left and right lateral CST in the PAM50 template space.^41^ Lesion volumes in both CSTs and CST volumes were extracted using *fslstats* and the lesion volume fractions calculated as


$$CST\;lesion\;volume\;fraction\;=\;\frac{Lesion\;volume\;in\;CST}{CST\;volume}\;\times \;100\;$$

These measures were extracted in the CST of the brain, the brainstem and the cervical SC (C1–C7), thoracic SC (T1–T10) or whole SC (C1–T10). Mean MTR values were extracted in the CST for the brain and the brainstem and three segments of the SC corresponding to the centre of the three acquired slabs (C4–C6, T4–T6 and T9–T10). For the sake of comparison, individual mean MP2RAGE qT1 values were extracted in the CST for the brain and the brainstem and the cervical SC (C4–C6).

Imaging and CMCT data were harmonized using ComBat in R (https://github.com/Jfortin1/neuroCombat_Rpackage; as of 18 June 2024). ComBat is a popular batch-effect correction tool based on empirical Bayes methods used in genomics^42^ and adapted for imaging^43^ to correct for variability between scanners. Each modality (CMCT, MTR, MP2RAGE and anatomical data) was processed independently in ComBat, with extracted measures in ROIs as input data, three biological covariates (age, sex and group) and parametric adjustments for model computation. In the Results section, harmonized measures for imaging and CMCT data are reported.

#### Imaging *z*-score maps and severity profiles

To evaluate the severity of the lesions, individual voxelwise *z*-score maps were created for MTR images. For this part, only data from Rennes were considered, since only seven HCs were available in Marseille, which would have led to inaccurate *z*-score maps. First, lesion masks and MTR maps were registered to MNI152 space for the brain and PAM50 for the SC using transformation parameters (see above). Mean and standard deviation MTR maps were calculated based on the HC group (*n* = 21). Then, individual patient *z*-score maps were computed as


$$z\;score=\;\frac{individual\;map\;-\;mean\;HC\;map}{standard\;deviation\;HC\;map}$$

**Figure 2:**
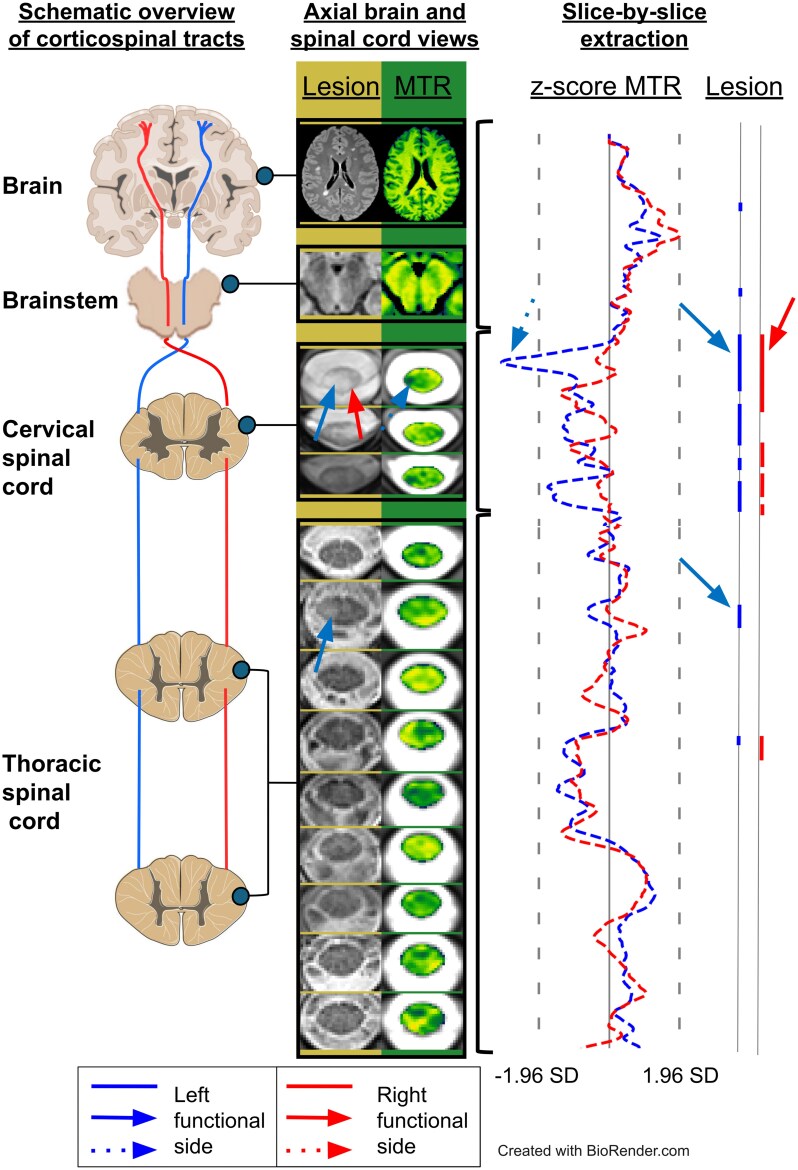
**Example of individual CST brain and SC profiles.** Clinical characteristics of the patient: female, 23 years old, EDSS score = 1.5, pyramidal FSS score = 1, ASIA motor score = 25 for each limb, right upper limb CMCT *z*-score = 2.1, left upper limb CMCT *z*-score = 7.7, right lower limb CMCT *z*-score = 0.5, left lower limb CMCT *z*-score = 7.6. Left part: schematic overview of the corticospinal tracts from the top of the brain to the thoracic SC. Central part: axial view of the lesions (yellow) and of the magnetization transfer ratio (green) in the brain and SC. Right part: MTR *z*-score profile (left) and location and extent of lesions based on conventional imaging (right; bold lines = lesions in the CST) along the whole corticospinal tract. In each part, red colour represents the right functional side, while blue colour represents the left functional side. Solid and dotted arrows point to lesion and MTR abnormalities, respectively. MT = magnetization transfer ratio; SD = standard deviation; CST = corticospinal tract. https://BioRender.com/tbwwxmu.

Based on registered individual lesion masks and *z*-score MTR maps masked by both left and right CSTs, individual profile graphs were generated, showing on the *y*-axis the slice level in the brain or SC, and on the *x*-axis the percentage of damaged CST in the slice and the mean MTR *z*-score in the corresponding slice. An example is provided in Fig. 2. These profiles allowed us to detect the lesions along the whole CST from the top of the brain to the bottom of the thoracic spine. Moreover, lesions in the CST were considered severe if the associated MTR *z*-map was below −1.96 SD (corresponding to *P* < 0.05).

The same profiles were repeatedly computed with qT1 *z*-score maps (and a thresholding at 1.96 SD) in addition to lesion and MTR *z*-score maps.

### Statistical analyses

The statistical analyses included three parts. First, descriptive analyses of the populations and associated clinical, electrophysiological and imaging scores were performed. Then, the associations between imaging and both electrophysiology and clinical scores were computed. Finally, the lesion severity was analysed. The first two sets of analyses were ROI based, while the severity was assessed with profiles along the whole CSTs (see descriptions above). All these analyses were performed with anatomical and MTR imaging and were replicated with qT1 as described below.

Statistical analyses were carried out in R (v. 4.4.0) using RStudio (v. 2022.07.2+576). Significance level was set to an alpha of 0.05. Regression analyses were corrected for multiple comparisons at *P* < 0.01 (0.05/5 clinical and electrophysiological measures). Lesion volume fractions were log transformed prior to all analyses to reduce skewness and normalize these measures. The R package *khroma*, which includes colour schemes specifically designed for colourblind people, was used to define the colours in all figures.^44^

#### Demographic statistics

Group differences between healthy participants and patients were assessed using two-sided two-sample *t*-tests for age and CMCT and using the chi-square test for sex. Partial correlations (Pearson) between mASIA score and CMCT per limb were computed with age, sex and disease duration as covariates.

#### Structural damage along the corticospinal tract in people with multiple sclerosis

Group differences between healthy participants and patients were assessed using two-sided two-sample *t*-tests (Welch approximation) for MTR in each ROI. Partial correlations (Pearson) between lesion loads and MTR in each ROI were computed with age, sex and disease duration as covariates. For this analysis, a correction for multiple comparisons (*P* < 0.05/10 ROIs = 0.005) was also reported.

#### Association between clinical scores and central motor conduction time and imaging

In the following, the functional side of the CST refers to the side of the CST functionally and anatomically related to each limb (e.g. for limbs on the right side of the body, the functional side refers to the left brain CST and the right SC CST). Both sides refer to the right and left CST sides together. Whole brain CST was considered for associations with all limbs. Whole SC CST was considered for association with the lower limbs, and the top of the SC CST to the end of the C7 vertebra level was considered for the association with upper limbs.

For each ROI and each functional side, regression models were computed between lesion volume fractions in each CST portion and EDSS, mASIA per limb and CMCT per limb. The same analyses were repeated with mean MTR values in each CST portion, age, sex and disease duration. Multivariable regression models were then computed with EDSS, upper or lower mASIA or upper or lower CMCT as dependent variables and all imaging measures and potential confounding factors with *P* < 0.2 at individual correlation as independent variables. Quantile–quantile plots, scale–location plots and residual versus leverage plots were visually checked. Variance inflation factors were computed to evaluate the collinearity between independent variables. For the sake of completeness, the same univariate and multivariable models were replicated for the other clinical measures (FSS, T25-FW, MSWS-12, 9HPT and qualiveen).

#### Impact of severe lesions on motor function

These analyses were performed in 70 functional sides (35 pwMS with two sides). Four types of analyses were conducted based on lesion severity using MTR *z*-score profiles (see above). First, the percentage of pwMS with at least one severe lesion was calculated per CST portion. Then, the impact of the severe lesion on motor function (mASIA and CMCT in upper and lower limbs) was determined using chi-square tests and the number of pwMS with non-severe or at least one severe lesion per subgroup of the normal/abnormal mASIA and CMCT in the upper and lower limbs. Third, logistic regressions were performed with CMCT status (normal/abnormal) as the dependent variable and lesion volume fraction in the brain and SC CST (separately) and presence or absence of at least one severe lesion in the functional side in the brain and SC CST (separately or together) as independent variables and potential confounding factors (age, sex, disease duration). Finally, association between previous motor relapse and severe lesion were described. Abnormality of CMCT per limb was defined after calculation of individual *z*-scores (based on HCs from Rennes) using the formula described above with the threshold of 1.96 (*P* < 0.05).

#### Additional analyses using quantitative T1

To evaluate the potential of using an alternative quantitative MRI sequence and to validate our results with another proxy of myelination, namely MP2RAGE, which can be acquired in the brain and cervical SC simultaneously (but does not cover the whole SC as MT) and is faster than MT (total acquisition time: ∼7 min versus ∼18 min, respectively), all analyses described above were replicated by replacing MTR with qT1.

## Results

### Population characteristics

The demographic and clinical characteristics of the 46 pwMS and 28 HCs included in the analyses are summarized in Table 1. The median [range] EDSS in pwMS was 2.5 [1.0–6.0], and the pyramidal FSS was 2.0 [1.0–3.0]. Twenty of the 46 pwMS had a motor deficit in at least one limb. mASIA score was abnormal for five of the 46 pwMS in at least one upper limb and 20 of the 46 pwMS in at least one lower limb. CMCT was higher in both upper (8.98 [3.18] versus 7.50 [1.88] ms, *P* = 0.003) and lower limbs (19.16 [7.09] versus 14.89 [3.07] ms, *P* < 0.001) in pwMS compared to HCs. A negative association was found between mASIA score and CMCT in the lower limbs (*r* = −0.315; *P* = 0.004), but not in the upper limbs (*r* = −0.142; *P* = 0.189). Positive associations were found between CMCT in the upper limbs and EDSS (*r* = 0.321; *P* = 0.002) and between CMCT in the lower limbs and EDSS (*r* = 0.233; *P* = 0.033).

**Table 1:** Participants’ demographic and clinical characteristics

Measure	HC (*n* = 28)	pwMS (*n* = 46)	HC versus pwMS, *P*-value
Age, years	39.01 (10.3)	40.42 (11.0)	0.582
Sex, % female	75.0	65.2	0.778
Disease duration, years		8.73 (5.56)	
EDSS, /10		2.5 [1.0–6.0]	
Pyramidal FSS, /6		2.0 [1.0–3.0]	
Nine-hole peg test (L/R combined), seconds		21.15 (5.3)	
Timed 25-foot walk, seconds		4.90 (1.0)	
ASIA motor upper limb, /25		25 [22–25]	
ASIA motor lower limb, /25		25 [10–25]	
CMCT upper limb, ms	7.50 (1.88)	8.98 (3.18)	0.003*
CMCT lower limb, ms	14.89 (3.07)	19.16 (7.09)	<0.001*
Normalised brain volume, L	1.54 (0.07)	1.56 (0.09)	0.45
SC cross-sectional area, mm^2^	64.6 (4.38)	60.7 (7.04)	0.01*

Data are shown as mean (± standard deviation) or median [range] for EDSS and ASIA motor scores. Two-sample *t*-tests were used to compare age between the HC and the pwMS, and χ^2^ was used for sex ratio comparisons. For ASIA and CMCT, data from the left and right sides were combined. Upper limb CMCT was missing for one pwMS and 13 HCs; lower limb CMCT was missing for two pwMS and 13 HCs; brain volume was missing for one pwMS and one HC. See Supplementary Material Text S2 for a description of the computation of brain volumes and SC cross-sectional areas.

EDSS = Expanded Disability Status Scale; ASIA = American Spinal Injury Association; CMCT = central motor conduction time.

^*^ *P* < 0.05.

### Structural damage along the corticospinal tract in people with multiple sclerosis

The imaging characteristics of the pwMS and HCs are summarized in Table 2.

**Table 2:** Participants’ CST imaging characteristics

		HC		pwMS		HC versus pwMS	
Measure	CST region of interest	Left	Right	Left	Right	*P*-value	Cohen’s *d*
Lesion volume fraction in % and lesion volume in mL	Brain			3.12 (7.37)	1.63 (2.82)		
				154.89 (375.53)	78.04 (134.39)		
	Brainstem			0.60 (1.62)	0.16 (0.44)		
				21.0 (56.62)	5.45 (14.73)		
	Cervical SC (C1−C7)			11.28 (17.69)	11.45 (16.23)		
				51.77 (80.21)	54.07 (77.30)		
	Thoracic SC (T1−T10)			4.78 (8.21)	4.72 (12.10)		
				25.75 (44.74)	25.86 (69.81)		
	Whole SC (C1−T10)			7.71 (11.28)	7.89 (13.08)		
				77.57 (113.37)	79.92 (137.15)		
MTR, in pu	Brain	42.817	43.563	42.438	43.531	0.196/0.895	0.28/0.03
		(0.948)	(0.837)	(1.556)	(1.212)		
	Brainstem	46.610	46.440	46.221	46.291	0.112/0.507	0.35/0.15
		(0.826)	(0.798)	(1.254)	(1.116)		
	Cervical SC (C4−C6)	42.716	43.022	40.965	40.912	**0.003** * **/** **<** 0.001*	0.62/0.77
		(1.395)	(1.730)	(3.435)	(3.220)		
	Thoracic SC (T4–T6)	41.164	41.005	38.669	38.702	**0.003** */0.025*	0.65/0.53
		(2.725)	(3.907)	(4.379)	(4.618)		
	Thoracic SC (T9–T10)	35.541	36.515	35.781	35.509	0.840/0.433	0.05/0.19
		(4.428)	(5.163)	(5.666)	(5.564)		

Data are shown as mean (± standard deviation) for lesion volume fraction and volume and as MTR in the CST of HCs and pwMS.

MTR = magnetization transfer ratio; pu = per unit.

^*^ *P* < 0.05. Bold values: *P* surviving to correction for multiple comparisons (*P* < 0.005).

### Corticospinal tract lesion load

Forty-five of the 46 pwMS (98%) had at least one lesion in their CSTs regardless of side (42/46 for the right CST and 41/46 for the left CST). Overall, the structural damage of the CST is predominantly located in the cervical portion of the SC. Thus, the mean lesion volume fraction was higher in the cervical portion of the CST than in the brain, brainstem or thoracic portion for both the left (11.28% versus 3.12%, 0.60% and 4.78%, respectively) and right sides (11.45% versus 1.63%, 0.16% and 4.72%, respectively).

### Corticospinal tract microstructure in ROI

Compared to the HCs, pwMS had significantly lower MTR values in the SC portion of the CST [lower MTR values in the cervical (C4–C6) and upper thoracic (T4–T6) CST portions for both the left and right sides (Table 2); all *P* < 0.05] but not in the brain and brainstem portions. Overall, MTR values were negatively associated with lesion load within the brain and SC portions of the CST (*P* < 0.05) but not in the brainstem portion (*P* > 0.4; see Supplementary Table S2).

### Association between corticospinal tract structural damage in ROI and functional consequences

#### Overall disability scores

Multivariable regressions with EDSS score as the dependent variable showed significant associations (*R*^2^ = 0.336), ordered by importance, with age, MTR in the upper thoracic SC (T4–T6) CST and lesion volume fraction in the brainstem CST (Table 3). No significant association was observed with the other clinical measures (FSS, T25-FW, MSWS-12, 9HPT and qualiveen) as dependent variables, except for the qualiveen test (*R*^2^ = 0.158), for which only an association with sex was observed.

**Table 3:** Associations between EDSS score and imaging (lesion and MTR)

		EDSS score			
		Univariate		Multivariable	
				Adj. *R*^2^ = 0.336, *P* < 0.001	
Measure	Functional CST region of interest	*r*	*P*	Stand. β coef	*P*
Lesion volume fraction	Brain	−0.016	0.882	NA	
	Brainstem	0.350	**<**0.001*	0.243	0.008*
	Cervical SC (C1–C7)	0.239	0.022*	0.103	0.452
	Thoracic SC (T1–T10)	0.240	0.021	0.126	0.313
MTR	Brain	−0.102	0.333	NA	
	Brainstem	−0.002	0.988	NA	
	Cervical SC (C4–C6)	−0.189	0.072	0.076	0.513
	Thoracic SC (T4–T6)	−0.341	**<**0.001*	−0.267	0.008*
	Thoracic SC (T9–T10)	−0.120	0.255	NA	
Age		0.304	0.003*	0.294	0.002*
Sex		−0.013	0.904	NA	
Disease duration		0.361	**<**0.001*	0.226	0.018

EDSS = Expanded Disability Status Scale; SC = spinal cord; MTR = magnetization transfer ratio; CST = corticospinal tract; Adj. *R*^2^ = adjusted *R*^2^; Stand. β coef = standardized β coefficient; NA = not applicable.

^*^ *P* corrected for multiple comparisons (corresponding to *P* < 0.01). Bold values: *P* not corrected.

#### Motor scores per limb

##### American Spinal Injury Association motor score per limb

No associations were observed between the mASIA score for any limb and any imaging modality (see Supplementary Table S3).

##### Central motor conduction time per limb

Multivariable regressions with CMCT score in the upper limbs as the dependent variable showed significant association (*R*^2^ = 0.608) with lesion volume fraction in the cervical SC and MTR in the cervical CST (Table 4). Multivariable regressions with CMCT score in the lower limbs as the dependent variable showed significant associations (*R*^2^ = 0.642), ordered by importance, with MTR in the brain, MTR in the thoracic SC CST, lesion volume fraction in the cervical SC CST, sex and disease duration.

**Table 4:** Associations between CMCT per limb and imaging (lesion and MTR)

		CMCT upper limb				CMCT lower limb			
		Univariate		Multivariable		Univariate		Multivariable	
				Adj. *R*^2^ = 0.608, *P* < 0.001				Adj. *R*^2^ = 0.642, *P* < 0.001	
Measure	Functional CST region of interest	*r*	*P*	Stand. β coef	*P*	*r*	*P*	Stand. β coef	*P*
Lesion volume fraction	Brain	0.133	0.211	NA		0.080	0.457	NA	
	Brainstem	0.199	0.060	0.020	0.774	0.288	0.006*	0.058	0.409
	Cervical SC (C1–C7)	0.747	**<0.001** *	0.540	**<**0.001*	0.637	**<0.001** *	0.344	0.001*
	Thoracic SC (T1–T10)	NA				0.426	**<**0.001*	−0.012	0.902
MTR	Brain	−0.387	**<**0.001*	−0.159	0.056	−0.503	**<0.001** *	−0.394	**<0.001** *
	Brainstem	−0.209	0.048	−0.056	0.481	−0.315	0.003*	0.064	0.453
	Cervical SC (C4–C6)	−0.577	**<0.001** *	−0.197	0.022*	−0.543	**<0.001** *	−0.071	0.433
	Thoracic SC (T4–T6)	NA				−0.513	**<0.001** *	−0.363	**<0.001** *
	Thoracic SC (T9–T10)	NA				−0.287	0.064	0.074	0.344
Age		−0.174	0.101	−0.015	0.835	0.042	0.696	NA	
Sex		−0.318	0.002*	−0.133	0.064	−0.243	0.021	−0.165	0.022*
Disease duration		−0.081	0.447	NA		0.208	0.051	0.144	0.040*

CMCT = central motor conduction time; SC = spinal cord; MTR = magnetization transfer ratio; Adj. *R*^2^ = adjusted *R*^2^; Stand. β coef = standardized β coefficient; NA = not applicable.

^*^ *P* corrected for multiple comparisons (corresponding to *P* < 0.01). Bold values: *P* not corrected.

### Frequency of severe lesions and impact on motor function

#### Corticospinal tract severe lesions

**Figure 3:**
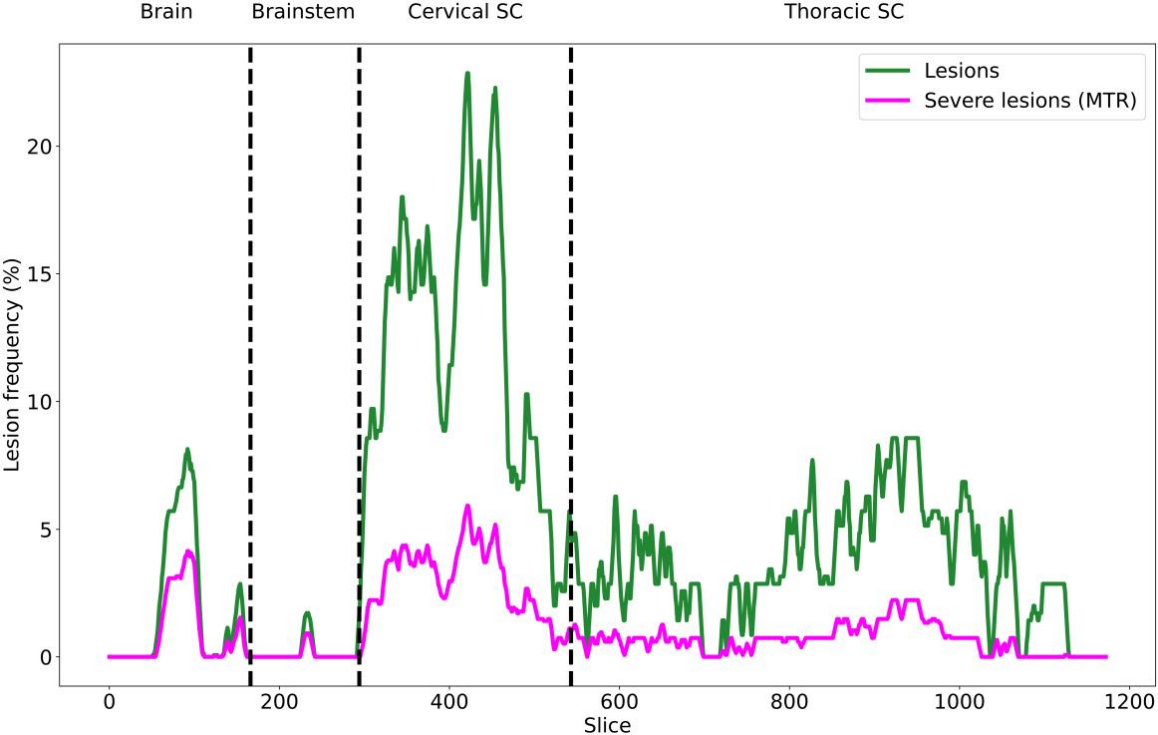
**Frequency of lesions (green) and severe lesions (red) in the brain, the brainstem and the cervical and thoracic SC in the 70 corticospinal tracts.** For each axial slice (1 mm slice thickness), the CST lesion frequency is represented by a solid line. The CST segments are demarcated by a dashed vertical line.

The right and left CST profiles of 35 pwMS from Rennes were analysed for a total of 70 CST profiles. An example of an individual CST profile from the top of the cortex to the bottom of the SC is provided in Fig. 2. Among the 70 CST profiles, 61 (87%) had at least one lesion and 43 (61.4%) at least one severe lesion. Overall, 84 of the 325 (25.8%) lesions were classified as severe. As illustrated in Fig. 3, the severe lesions were mainly located in the SC portion of the CST (66.7% versus 33.3% for the brain portion), especially in the cervical SC (41.7% versus 25% in the thoracic SC). The areas considered abnormal (MTR > 1.96 SD) were mainly located in segmented lesions (48 of the 57 abnormal *z*-score areas, 84.2%).

#### Severe corticospinal tract lesions and extra-lesional damage along the tract

In 43 of the 70 CSTs with at least one severe lesion, the mean extra-lesional MTR was lower than in the 27 CSTs without a severe lesion, both in the brain (43.7 [1.2] versus 44.5 [0.9], *P* = 0.004) and in the SC SCT (38.3 [3.2] versus 40.7 [1.7], *P* < 0.001).

#### Association between severe lesions and motor function

**Figure 4:**
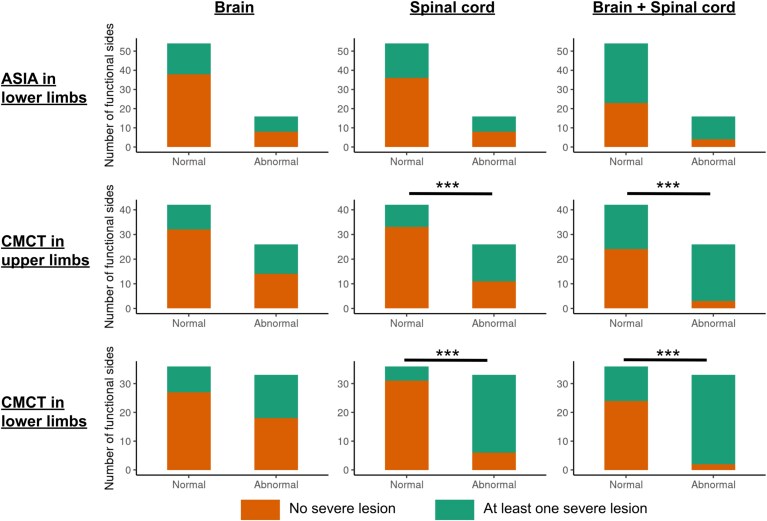
**Number of functional sides of pwMS (*n* = 70 corresponding to 35 patients with two sides) having no severe lesion (orange) or at least one severe lesion (green) in the brain (left column), the SC (middle column) or both together (right column) in subgroups based on the ASIA motor score in the lower limbs (top line) and CMCT in the upper (middle line) and lower limbs (bottom line).** The difference in the ratio of the number of functional sides in pwMS having at least one severe lesion in both subgroups (normal/abnormal based on ASIA motor score or CMCT) was evaluated using chi-square tests. CMCT = central motor conduction time. **P < 0.01; ***P < 0.001.

As illustrated in Fig. 4, severe lesions were more frequent in profiles associated with abnormal CMCT compared to normal CMCT, in particular when both brain and SC CST portions were considered (severe lesions in normal/abnormal CMCT in the upper limbs: 42.9%/88.5%; in the lower limbs: 33.3%/93.9%; all *P* < 0.001). The same results were not observed for lower limb mASIA (only three pwMS had abnormal upper limb mASIA and were not considered for these analyses). In three pwMS with abnormal upper limb CMCT, however, no severe CST lesion was found. Similarly, in two pwMS with abnormal lower limb CMCT, no severe CST lesion was found either in the brain or in the SC.

When both lesion load and lesion severity were taken into account in multivariable logistic regression models (Supplementary Table S4), the lesion volume fractions in the cervical SC CST (OR [95% CI]: 1.87 [1.44–2.44], *P* < 0.001) and in the brain (OR [95% CI]: 1.44 [1.15–1.81], *P* = 0.002) were the explanatory variables associated with an increased risk of having an abnormal CMCT in the upper limbs, and the presence of a severe lesion in the SC CST (OR [95% CI]: 1.74 [1.36–2.24], *P* < 0.001) was the only explanatory variable associated with an increased risk of having an abnormal lower limb CMCT. The areas under the curve of the global models were 0.943 and 0.924, respectively.

#### Association between previous motor relapse and severe lesion

A detailed history of MS, with regular clinical assessments since the first relapse, was available for 17 of the 35 patients included in the severe lesion analysis. Among these patients, 14 had experienced a previous motor relapse, responsible for a motor deficit. A severe lesion was identified on the motor tract on the side corresponding to the relapse in 11 of the 14 patients (79%). However, in nine of the 17 patients (53%), a severe lesion on the motor tract was also identified on a side without history of motor relapse during follow-up. In six of the nine cases, the CMCT was abnormal in the corresponding side.

### Additional analyses using quantitative T1

MTR and qT1 were negatively associated in all portions (*P* < 0.05; Supplementary Table S2). In the multivariable models, EDSS score was associated with lesion volume fraction in the brainstem CST and with age, and the upper limb mASIA score was associated with qT1 in cervical SC and upper and lower limb CMCT with lesion volume fraction in cervical SC (Supplementary Tables S5–S8).

As illustrated in Supplementary Fig. S2, severe lesions were more frequent in profiles associated with abnormal CMCT than normal CMCT, in particular when both brain and SC CST portions were considered. The multivariable logistic regression model revealed that the lesion volume ratio in the SC (OR [95% CI]: 1.66 [1.27–2.16], *P* < 0.001) was the only explanatory variable associated with an increased risk of having an abnormal lower limb CMCT {severe lesion in cervical SC [excluding the thoracic part] did not survive to multiple comparisons [OR (95% CI): 1.31 (1.00–1.71), *P* = 0.048]}. The area under the curve of the global model was 0.91. The same results as for MTR were found for multivariable logistic regression models with lower limb mASIA or upper limb CMCT as dependent variables (Supplementary Table S9).

## Discussion

In this study, we combined conventional and quantitative MRI to assess lesion load and myelin content along the whole motor pathway, from the brain to the lower SC. We showed that lesions on the CST are more frequent than functional consequences measured by electrophysiology and by clinical assessment of motor deficits. We described various degrees of lesion myelin content along the tract, with frequent severe lesions in the cervical SC CST. We found associations between lesion severity and abnormal CMCT measurements and between lesion severity and extra-lesional damage along the tract. In contrast, no association could be demonstrated between the mASIA score for a limb and the corresponding CST lesion load/severity, illustrating the complex relationship between focal lesion, motor conduction time and its translation into a motor deficit accurately measurable by clinical test. Finally, the main results obtained with MTR were replicated with the faster qT1.

### Frequency of corticospinal tract lesions and functional consequences

Firstly, in our cohort, all patients but one (98%) had lesions on motor tracts, with a higher lesion volume fraction in the SC portion of the CST compared to the brain portion, especially at the cervical level. This repartition of lesions along the CST is in line with the literature^5-7,45,46^ as well as the high frequency of lesions in the CST in pwMS.^6^ Secondly, CST lesions were more frequent than electrophysiological consequences, underlining the fact that not all lesions have the same functional consequences. A similar result was recently reported in a cohort of early relapsing–remitting multiple sclerosis patients, in which tibial nerve somatosensory evoked potentials were less frequently abnormal than SC MRI (22% versus 68%).^47^ Thirdly, CST lesions were also much more frequent in our cohort than the clinical motor deficits measured using the mASIA score, and only a weak association was observed between the mASIA and the CMCT on the corresponding limb.

The specific characteristics of the mASIA and CMCT measures need to be discussed in order to interpret these results. CMCT is a continuous value that reflects CST myelin damage, whereas the axonal loss plays an important role in persistent deficits.^22^ Similarly, visual evoked potentials (VEPs) usually remain altered after optic neuritis, even though visual acuity has returned to normal.^48^ Interestingly, in several longitudinal cohorts of multiple sclerosis patients, multimodal Eps, including MEPs, have been predictive of EDSS increase during follow-up but were not or only moderately associated with baseline EDSS.^49,50^ Sensitivity of EPs to subclinical tract damage can explain the association between EPs and later EDSS when compensatory mechanisms fail.^51^ This sensitivity of MEPs to subclinical motor pathway involvement is well illustrated in our study by the moderate to strong associations between CST imaging metrics (both lesion load and MTR, especially at the SC level) and CMCT. This cohort will need to be followed longitudinally to determine whether a relationship emerges between CMCT measurements and the subsequent development of motor deficits in the corresponding limb.

The lack of a strong relationship between lesion load and motor deficit, and between CMCT and motor deficit in our cohort, may, however, be attributed not only to its cross-sectional design but also to the mASIA score. The performance of this score, developed in patients with SC injury, has not been formally evaluated in pwMS, even though it has already been used in this population.^52^ We chose this score because it has the advantage of evaluating motor impairment for each limb. It relies on a manual strength testing of five muscles in the upper limbs and five in the lower limbs according to the MRC scale. However, it lacks sensitivity, especially in the higher grade values.^53^ Furthermore, Grade 4 encompasses a wide range of strength reduction and does not distinguish the different degree of strength reduction in this range.^54,55^ In our study, patients mostly had higher Grades 4 and 5. Moreover, we cannot exclude false impairment ratings, such as sensitivity deficit, cerebellar hypotonia or pain, which are common symptoms in multiple sclerosis and can mimic a muscle weakness. Ultimately, the electrophysiological measures (MEPs) used in this study likely provide a more objective and quantifiable assessment of upper motor neuron involvement.

### Individual profiles of microstructural damage along the corticospinal tract and presence of at least a severe lesion

In this study, we wanted to test specifically the association between the presence of a severe lesion in the CST and the occurrence of motor deficits and/or prolonged CMCT as suggested by the concept of ‘critical lesion’, previously reported in progressive multiple sclerosis patients.^3,4^ The ‘critical lesion’ is based on a visual appreciation on anatomical images, and defined as lesions located along the CST, associated with focal atrophy on MRI—thus suggesting a certain degree of severity—and consistent with the patient’s clinical presentation in terms of the expected side and limb involvement. Here, we went further by developing the concept of a severe lesion based on quantitative imaging and statistical analyses using *z*-score compared to a healthy population. This method is thus potentially more objective for detecting focal lesions with substantial microstructural damage and could be applied earlier, in patients with a relapsing phenotype of the disease who do not show visible SC atrophy at the level of the lesion and progressive motor deficit. One of its limitations, however, is that it relies on the choice of a statistical threshold.

Interestingly, the presence of a severe lesion in the CST was associated with more pronounced extra-lesional damage assessed using the MTR *z*-score along the corresponding tract. From a pathophysiological point of view, this observation may indicate an alteration of the microstructure, propagating anterogradely and/or retrogradely along the tract, measurable even in pwRRMS.^56^ We also explore the association between the lesion severity and their functional consequences. Severe lesions in the CST were much more common in patients with prolonged CMCT in the corresponding limb compared with those with non-prolonged CMCT (33.3% versus 93.9% for lower-limb CMCT). Moreover, the presence of a severe lesion in the SC CST was the only explanatory variable associated with an increased risk of having an abnormal lower-limb CMCT. Overall, these results tend to confirm our initial hypothesis concerning the key role of severe lesions. As in the ROI analysis, however, no significant association was found between the presence of at least a severe lesion in the CST tract and the concurrent motor deficit in the corresponding limb assessed using the mASIA score. Similarly, it is important to follow this cohort over the long term to explore whether a link appears (i.e. whether some patients convert to a progressive form of the disease involving the corresponding limb). Interestingly, severe lesions of the CST (associated most of the time with prolonged CMCT) were found in 53% of patients with no history of motor relapse on the corresponding side. This observation suggests that even severe lesions in the CST may be clinically asymptomatic in patients with RRMS. The question of the development kinetics of severe CST lesions remains to be clarified: do they result from the progressive worsening of initially mild subclinical lesions?

### Strengths and limitations

The main strengths of this study are its prospective design, the inclusion of a homogeneous cohort of RRMS patients with motor deficits or hyperreflexia at clinical examination, the association of conventional and two quantitative MRI acquisitions to approximate myelin content from the motor cortex to the lower part of the SC and the evaluation of motor function using both detailed clinical examination and MEP. These features provide a unique dataset with which to study the consequences on motor function of the severity of demyelination along the CST beyond any focal lesions detected on conventional MRI.

The main limitation of our study, as previously discussed, relates to the mASIA score used to evaluate motor disability. One way to alleviate these limits is the use of a dynamometer to evaluate motor impairment of a limb.^14^ Similarly, the presence of a Babinski sign and/or hyperreflexia, which were part of the inclusion criteria, is known to be associated with intra- and inter-observer variability.^57^ Secondly, our cohort only includes relapsing–remitting multiple sclerosis patients. A similar study dedicated to progressive pwMS would allow exploration of the relationship between a severe lesion on a CST and the progressive motor impairment that often affects one limb in pwMS. Finally, not all patients with prolonged CMCT had a severe lesion. These results need to be interpreted in the light of several limitations concerning MTR profiles. Indeed, MTR maps were less reliable at the bottom of the SC thoracic part, as illustrated in Table 2 (high MTR SD values at the thoracic levels in HCs). Moreover, lesions were identified by manual segmentation in the SC. This type of segmentation has significant intra- and inter-operator variability in both lesion detection and edge demarcation, which can lead to unseen or partially missing lesions.^58^

## Conclusion

In this study, we reconstructed the CST from the brain to the lower SC, quantified its lesion load and assessed the severity of each lesion along the tract. We identified varying levels of microstructural damage within CST lesions and found associations between lesion severity and both extra-lesional damage and CMCT measurements. We can hypothesize that severe lesions along the CST—particularly at the cervical level—play a key role in subsequent pathway degeneration and likely contribute significantly to functional impairment later in the disease course. Long-term follow-up of this cohort will be essential to validate this hypothesis. When transposed to clinical practice, these observations underscore the importance of preventing new lesions—whether they are immediately severe or may become severe over time—through the early initiation of effective disease-modifying treatments as well as the importance of developing therapies that promote remyelination and neuroprotection and to evaluate their effects at the cervical SC level.

## Supplementary Material

fcag140_Supplementary_Data

## Data Availability

Data supporting the findings of this study are available from the corresponding author, upon reasonable request, on osf.io.
