# Supplementary material for: Motor tract lesion mapping from the brain to the lower spinal cord in people with relapsing–remitting multiple sclerosis: exploring the association between lesion severity and functional consequences by limb
Source: Brain Commun. 2026 Apr 17;8(3):fcag140. doi: 10.1093/braincomms/fcag140 (PMC13184689; doi:10.1093/braincomms/fcag140)
Supplement: fcag140_Supplementary_Data [file fcag140_supplementary_data.pdf]

Motor tract lesion mapping from brain to lower spinal cord in people with relapsing remitting multiple sclerosis: Exploring the association between lesion severity and functional consequences by limb

Authors and affiliations

Malo Gaubert,[1,2,†] Alice Dufey,[3,†] Elise Bannier,[1,2] Benoît Combès,[2] Audrey Rico,[4,5,6] Jean-Christophe Ferré,[1,2] Raphaël Chouteau,[3] Paul Sauleau,[7] Guillaume Hamon,[1] Mathilde Liffran,[2] Laure Michel,[3] Emmanuelle Le Page,[3] Virginie Callot,[4,5] Bertrand Audoin,[4,5,6] Sarah Demortière,[4,5,6] Anne Kerbrat[2,3]

[†] Malo Gaubert and Alice Dufey contributed equally to this work.

1 Univ Rennes, CHU Rennes, (Service de radiologie et imagerie médicale), F-35000 Rennes, France

2 Univ Rennes, Inria, CNRS, Inserm, IRISA UMR 6074, Empenn U1228, Rennes, France

3 Univ Rennes, CHU Rennes, (Service de neurologie), F-35000 Rennes, France

4 Assistance Publique-Hopitaux de Marseille (AP-HM), Hôpital Universitaire Timone, CEMEREM, Marseille, France

5 Aix-Marseille University, CNRS, CRMBM, Marseille, France

6 Department of Neurology, Assistance Publique-Hopitaux de Marseille (AP-HM), Hôpital Universitaire Timone, Marseille, France

7 Univ Rennes, CHU Rennes, (Service des explorations fonctionnelles), F-35000 Rennes, France

Correspondence to: Anne Kerbrat

Univ Rennes, CHU Rennes, (Service de neurologie), 2 rue Henri Le Guilloux, F-35000 Rennes, France

anne.kerbrat@chu-rennes.fr

Table of Contents

Supplementary Figure S1: Study flow-chart 3

Supplementary Text S1: Neurophysiological recordings and measurements 4

Supplementary Text S2: Processing of anatomical images 5

Supplementary Table S1: MRI acquisition parameters 6

Supplementary Table S2: Associations between the different imaging modalities 7

Supplementary Table S3: Associations between ASIA score per limb and imaging (lesion and MTR) 8

Supplementary Table S4: Logistic regressions between ASIA and CMCT per limb and imaging (lesion and MTR) 9

Supplementary Table S5: Participant's CST Imaging Characteristics (qT1) 10

Supplementary Table S6: Associations between EDSS score and imaging (lesion and qT1) 11

Supplementary Table S7: Associations between ASIA per limb and imaging (lesion and qT1) 12

Supplementary Table S8: *Associations between CMCT per limb and imaging (lesion and qT1)* 13

Supplementary Table S9: *Logistic regressions between ASIA and CMCT per limb and imaging (lesion and qT1)* 14

Supplementary Figure S2: According to MP2RAGE, Number of functional sides of people with multiple sclerosis (n = 70 corresponding to 35 patients with two sides) having no severe lesion (green) or at least one severe lesion (orange) in the brain (left column), the spinal cord (middle column) or both gathered (right column) in subgroups based on ASIA in lower limbs (top line), CMCT in upper (middle line) and lower limbs (bottom line). 14

Supplementary Figure S1: Study flow-chart

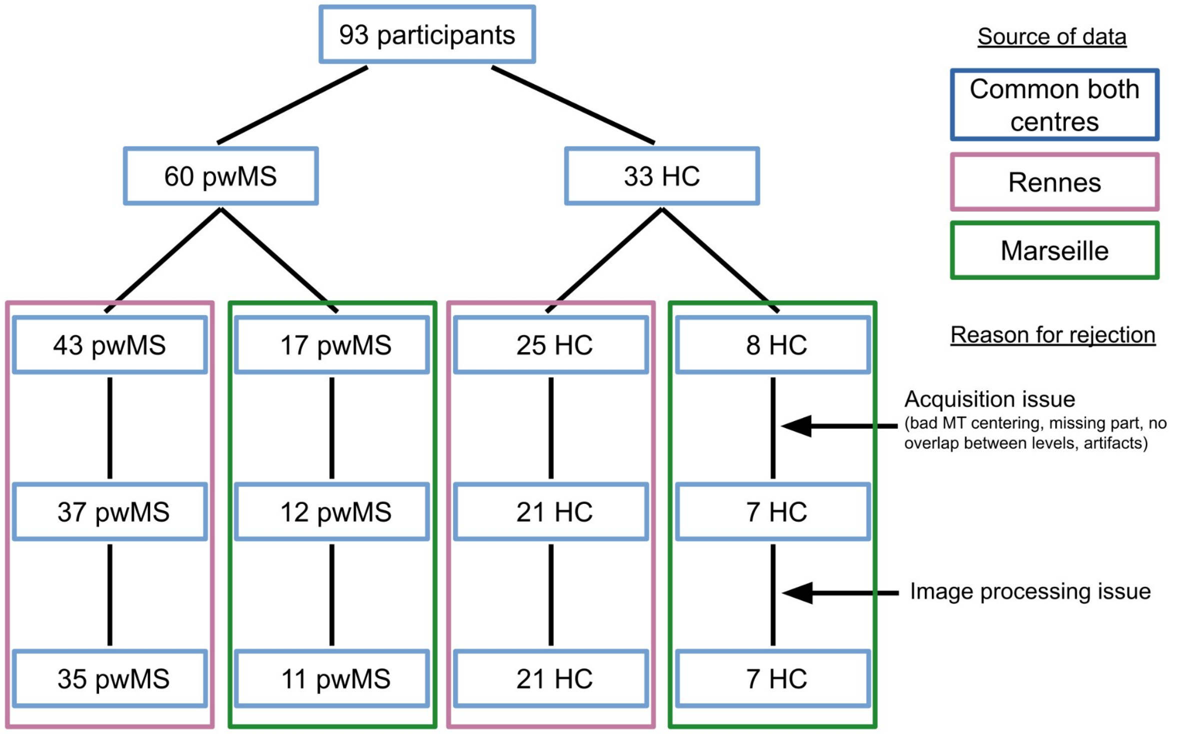

Legend: pwMS = person with multiple sclerosis; HC = healthy control; MT = magnetisation transfer.

Supplementary Text S1: Neurophysiological recordings and measurements

Electrophysiological parameters were recorded using a Nicolet EDX apparatus (Natus Medical, Madison, Wisconsin, USA). A Magstim 200 Mono Pulse stimulator (maximum output 2.0 T) with a circular coil (Magstim Company, Whitland, Wales, UK) was used for Transcranial Magnetic Stimulation (TMS). MEPs were recorded on the abductor digiti minimi muscle for upper limbs and on the tibialis anterior for lower limbs using surface electrodes. For TMS, the coil was placed over the vertex and moved slightly in all directions until the position providing the largest response was found. Stimuli started at 30% of the maximum intensity delivered by the stimulator then progressive intensity increase was used in order to determine the resting motor threshold. MEPs are measured at a resting motor threshold of 140%. Stimulations of the motor root were applied over C7-T1 vertebrae level for the upper limbs and over lumbar L1 vertebra level for the lower limbs. Central motor conduction time (CMCT) was calculated with motor root stimulation technique using the formula: cortical MEP latency - CMAP produced by electromagnetic stimulation over the spinal segment latency.

Supplementary Text S2: Processing of anatomical images

Brain volumes and spinal cord cross-sectional (CSA) area were both calculated based on MPRAGE T1w images. Brain anatomical measures were computed using the SIENAX tool included in FSL. Notably, we used in our statistical analyses the whole brain volume, normalised for skull size. CSA were computed using the SCT toolbox combining the vertebra C2 and C3 levels after spinal cord segmentation (*sct_deepseg*). Whole brain volumes and CSA were included in a ComBat model to account for scanner variability between the two centres, with three biological covariates (age, sex and disease diagnosis) and parametric adjustments for model computation.

Supplementary Table S1: MRI acquisition parameters

	Acquisition parameters								
	**Acquisition time (min)**	**Number of acquisitions**	**Orientation**	**Voxel size (mm^3)**	**TR (ms)**	**TE (ms)**	**TI (ms)**	**Flip angle (°)**	**Matrix size (voxel)**
**3D T1 MPRAGE brain**	4:26	1	sagittal	1×1×1	1900	2.26	900	9	256×256×176
**3D FLAIR brain**	5:17	1	sagittal	1×1×1	7000	413	2100	120	240×256×176
**3D MT0 brain**	4:45	1	transversal	1×1×3	43	3.13	NA	9	224×224×44
**3D MT1 brain**	4:45	1	transversal	1×1×3	43	3.13	NA	9	224×224×44
**3D MP2RAGE brain & SC**	7:18	1	sagittal	0.9×0.9×1	4000	2.48	TI1/TI2= 650/2000	FA1/FA2= 4/5	260×260×176
**2D T2w SC**	4:07	2: upper and lower parts of the SC	sagittal	0.5×0.5×2.5	3900	81	NA	160	512×512×17
**2D T2 star SC**	3:59	2: C1-C3 and C4-C7	transversal	0.4×0.4×3.0	849	23	NA	30	512×512×20
**2D T2w SC**	4:15	2: C7-T8 and T8-L1	transversal	0.2×0.2×3.0	4530	47	NA	160	768×768×60
**2D T2 STIR SC**	3:51	1	sagittal	0.3×0.3×3.0	3700	47	230	160	640×640×15
**3D MT0 SC**	4:07	3: centered on C5,T5 and T10-T11	transversal	0.4×0.4×5.0	43	3.13	NA	9	512×512×36
**3D MT1 SC**	4:07	3: same as 3D MT0 SC	transversal	0.4×0.4×5.0	43	3.13	NA	9	512×512×36

Supplementary Table S2: Associations between the different imaging modalities

			Left side		Right side	
Cohort	Measures	Region of interest (CST in the)	R	p	R	p
pwMS only	Lesion volume ratio × MTR	Brain	−.674	<.001*	−.381	.012*
		Brainstem	−.122	0.436	.033	0.836
		Cervical SC	−.676	<.001*	−.522	<.001*
		Thoracic SC (T1T10 x T4T6)	−.330	.031*	−.344	.024*
		Thoracic SC (T1T10 x T9T10)	−.257	0.096	−.543	<.001*
	Lesion volume ratio × qT1	Brain	.804	<.001*	.606	<.001*
		Brainstem	.176	0.265	−.079	0.618
		Cervical SC	.664	<.001*	.760	<.001*
	MTR × qT1	Brain	−.651	<.001*	−.323	.037*
		Brainstem	−.590	<.001*	−.596	<.001*
		Cervical SC	−.738	<.001*	−.794	<.001*
HC + pwMS	MTR × qT1	Brain	−.651	<.001*	−.323	.037*
		Brainstem	−.590	<.001*	−.596	<.001*
		Cervical SC	−.738	<.001*	−.794	<.001*

*Legend: pwMS: people with Multiple Sclerosis; HC: healthy controls; SC: spinal cord; MTR: magnetisation transfer ratio; qT1: MP2RAGE quantitative T1. Age, sex and disease duration were added as covariates in the models. * p < .05.*

Supplementary Table S3: Associations between ASIA score per limb and imaging (lesion and MTR)

		**ASIA Upper limb**				**ASIA Lower limb**			
		**Univariate**		**Multivariable** Adj. R^2: .017, p: .170		**Univariate**		**Multivariable** Adj. R^2: .049, p: .060	
**Measure**	**Functional CST region of interest**	**r**	**p**	**Stand. β coef**	**p**	**r**	**p**	**Stand. β coef**	**p**
Lesion volume fraction	Brain	.028	.787			−.006	.955		
	Brainstem	.070	.506			−.104	.324		
	Cervical SC (C1C7)	−.101	.337			−.064	.544		
	Thoracic SC (T1T10)					−.129	.221		
MTR	Brain	.111	.294			.083	.429		
	Brainstem	−.015	.884			.072	.494		
	Cervical SC (C4C6)	.187	.074			.098	.353		
	Thoracic SC (T4T6)					.135	.198		
	Thoracic SC (T9T10)					.057	.591		
Age		−.053	.613			−.209	.046		
Sex		−.159	.130			−.144	.171		
Disease duration		−.086	.412			−.004	.973		

Legend: ASIA: American spinal injury association; SC: spinal cord; MTR: magnetisation transfer ratio; Adj. R^2: adjusted R^2; Stand. β coef: standardised β coefficient. * p *corrected for multiple comparisons (corresponding to a* p < *.01).*

Supplementary Table S4: Logistic regressions between ASIA and CMCT per limb and imaging (lesion and MTR)

		**ASIA Lower limb**		**CMCT Upper limb**		**CMCT Lower limb**	
		Adj. R^2: .135<br>*p*: .023<br>AUC: .787		Adj. R^2: .473<br>*p* < .001<br>AUC: .943		Adj. R^2: .516<br>*p* < .001<br>AUC: .924	
**Measure**	**Functional CST region of interest**	**Stand. β coef**	***p***	**r**	***p***	**Stand. β coef**	***p***
Lesion volume fraction	Brain	.446	**.003***	.367	**.002***	.273	.014
	Whole/Cervical SC	.303	.069	.628	**<.001***	.311	.015
MTR	Brain	−.074	.613	−.152	.195	−.016	.882
	Cervical SC	−.152	.364	.033	.774	.558	**<.001***
Age		.171	.201	.076	.484	.123	.203
Sex		−.203	.876	−.173	.101	.082	.398
Disease duration		.255	.037	−.119	.208	.003	.973

Legend: ASIA: American spinal injury association; CMCT: central motor conduction time; SC: spinal cord; qT1: quantitative MP2RAGE; Adj. R^2: adjusted R^2; Stand. β coef: standardised β coefficient; AUC = area under the curve. Age, sex and disease duration were added in the models as covariates. * p *corrected for multiple comparisons (corresponding to a* p < *.01).*

Supplementary Table S5: Participant's CST Imaging Characteristics (qT1)

		HC		pwMS		HC vs. pwMS	
**Measure**	**Functional CST region of interest**	**Left**	**Right**	**Left**	**Right**	***p*-value**	**Cohen's d**
qT1, in ms	Brain	838.44 (21.60)	822.73 (22.49)	872.92 (49.62)	843.77 (28.67)	<.001* / .001*	.84 / .79
	Brainstem	924.79 (20.83)	920.81 (19.45)	944.05 (37.76)	930.04 (33.39)	.007* / .141	0.59 / 0.32
	Cervical spinal cord (C4C6)	916.09 (38.82)	909.73 (35.13)	995.77 (82.04)	996.32 (92.91)	<.001* / <.001*	1.15 / 1.13

Legend: Data are shown as means (± standard deviation) for MP2RAGE qT1 in the CST of healthy controls (HC) and people with multiple sclerosis (pwMS). Legend: qT1 = MP2RAGE quantitative T1, ms = millisecond. * p *< .05.*

Supplementary Table S6: Associations between EDSS score and imaging (lesion and qT1)

		EDSS score			
		Univariate		Multivariable	
				Adj. R^2: .297, *p*: <.001,	
**Measure**	**Functional CST in**	**r**	**p**	**Stand. β coef**	**p**
Lesion volume fraction	Brain	−.016	.881		
	Brainstem	.350	**<.001***	.270	**.004***
	Cervical SC (C1C7)	.239	.022	.022	.880
	Thoracic SC (T1T10)	.240	.021	.107	.435
qT1	Brain	−.024	.824		
	Brainstem	−.027	.801		
	Cervical SC (C4C6)	.329	**.001***	.172	.240
Age		.304	**.003***	.276	**.005***
Sex		−.013	.904		
Disease duration		.361	**<.001***	.216	.035

Legend. EDSS: expanded disability status scale; SC: spinal cord; qT1: MP2RAGE quantitative T1; Adj. R^2: adjusted R^2; Stand. β coef: standardised β coefficient. Age, sex and disease duration were added in the models as covariates. * p *corrected for multiple comparisons (corresponding to a* p *< .01).*

Supplementary Table S7: Associations between ASIA per limb and imaging (lesion and qT1)

		ASIA Upper limb				ASIA Lower limb			
		Univariate		Multivariate<br>Adj. R^2: .085<br>p: .007		Univariate		Multivariate<br>Adj. R^2: .078,<br>p: .017	
**Measure**	**Functional CST region of interest**	**r**	**p**	**Stand. β coef**	**p**	**r**	**p**	**Stand. β coef**	**p**
Lesion volume fraction	Brain	.028	.787			−.006	.955		
	Brainstem	.070	.506			−.104	.324		
	Cervical SC (C1C7)	−.101	.337			−.064	.544		
	Thoracic SC (T1T10)					−.129	.221		
qT1	Brain	.021	.841			−.050	.639		
	Brainstem	.003	.974			−.020	.848		
	Cervical SC (C4C6)	−.232	.026	−.291	**.006***	−.175	.095	−.233	.028
Age		−.053	.613			−.209	.046	−.201	.050
Sex		−.159	.1303	−.233	.027	−.144	.171	−.179	.092
Disease duration		−.086	.412			.004	.973		

Legend: ASIA: American spinal injury association; SC: spinal cord; qT1: MP2RAGE quantitative T1; Adj. R^2: adjusted R^2; Stand. β coef: standardised β coefficient. Age, sex and disease duration were added in the models as covariates. * p *corrected for multiple comparisons (corresponding to a* $p < .01$*).*

Supplementary Table S8: *Associations between CMCT per limb and imaging (lesion and qT1)*

		CMCT Upper limb				CMCT Lower limb			
		Univariate		Multivariable<br>Adj. R^2: .546, *p*: <.001		Univariate		Multivariable<br>Adj. R^2: .449, *p*: <.001	
**Measure**	**Functional CST region of interest**	**r**	***p***	**Stand. β coef**	***p***	**r**	***p***	**Stand. β coef**	***p***
Lesion volume fraction	Brain	.133	.211			.080	.457		
	Brainstem	.199	.060	.035	.645	.288	**.006***	−.129	.137
	Cervical SC (C1C7)	.747	**< .001***	.669	**<.001***	.637	**< .001***	.481	**<.001***
	Thoracic SC (T1T10)					.426	**<.001***	−.026	.835
qT1	Brain	.013	.901			.078	.475		
	Brainstem	.198	.064	.063	.425	.330	**.002***	.191	.030
	Cervical SC (C4C6)	.590	**< .001***	.054	.623	.567	**< .001***	.140	.303
Age		−.174	.101	.016	.837	.042	.696		
Sex		−.318	**.002***	−.095	.219	−.243	.022	−.039	.656
Disease duration		−.081	.447			.208	.051	.067	.465

Legend: CMCT: central motor conduction time; SC: spinal cord; qT1: MP2RAGE quantitative T1; Adj. R^2: adjusted R^2; Stand. β coef: standardised β coefficient. Age, sex and disease duration were added in the models as covariates. * p *corrected for multiple comparisons (corresponding to a* p < *.01).*

Supplementary Table S9: *Logistic regressions between ASIA and CMCT per limb and imaging (lesion and qT1)*

		ASIA Lower limb		CMCT Upper limb		CMCT Lower limb	
		Adj. R^2: .156<br>*p*: .019<br>AUC: .806		Adj. R^2: .481<br>*p* < .001<br>AUC: .940		Adj. R^2: .396<br>*p* < .001<br>AUC: .906	
**Measure**	**Functional CST region of interest**	**Stand. β coef**	***p***	**r**	***p***	**Stand. β coef**	***p***
Lesion volume fraction	Brain	.506	**<.001***	.388	**.001***	.203	.101
	Whole/Cervical SC	.228	.150	.573	**<.001***	.506	**<.001***
qT1	Brain	−.221	.134	−.116	.323	.099	.425
	Cervical SC	.132	.407	.079	.541	.268	.048
Age		.022	.868	.096	.376	.189	.096
Sex		.072	.598	−.178	.116	.099	.397
Disease duration		.228	.083	−.128	.219	.062	.576

Legend. ASIA: American spinal injury association; CMCT: central motor conduction time; SC: spinal cord; qT1: MP2RAGE quantitative T1; Adj. R^2: adjusted R^2; Stand. β coef: standardised β coefficient. Age, sex and disease duration were added in the models as covariates. * p *corrected for multiple comparisons (corresponding to a* p < *.01).*

Supplementary Figure S2: According to MP2RAGE, Number of functional sides of people with multiple sclerosis (n = 70 corresponding to 35 patients with two sides) having no severe lesion (green) or at least one severe lesion (orange) in the brain (left column), the spinal cord (middle column) or both gathered (right column) in subgroups based on ASIA in lower limbs (top line), CMCT in upper (middle line) and lower limbs (bottom line).

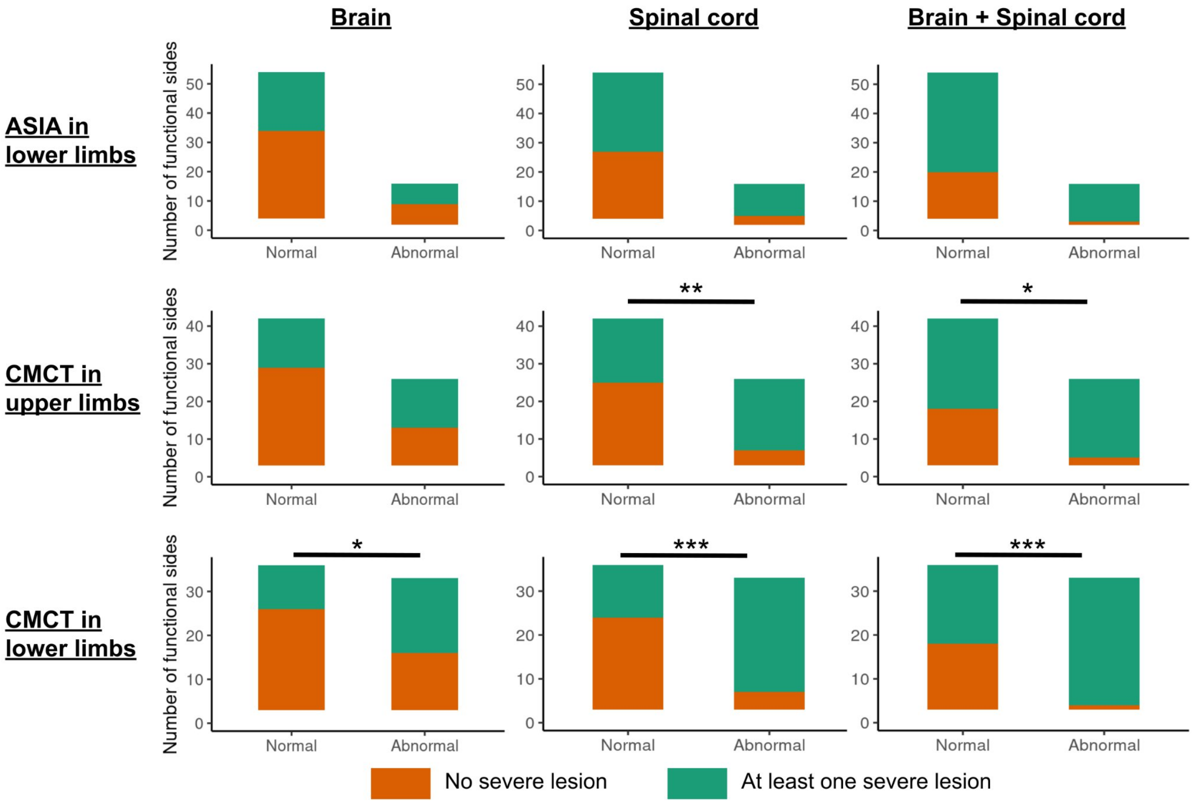

*The difference of ratio of the number of functional sides in pwMS having at least one severe lesion in both subgroups (normal/abnormal based on ASIA or CMCT) was evaluated using Chi² tests. Legend: CMCT: central motor conduction time. * p<.05; ** p<.01; *** p<.001. Number of functional sides evaluated = 70.*
